# TEK and biodiversity management in agroforestry systems of different socio-ecological contexts of the Tehuacán Valley

**DOI:** 10.1186/s13002-016-0102-2

**Published:** 2016-07-22

**Authors:** Mariana Vallejo-Ramos, Ana I. Moreno-Calles, Alejandro Casas

**Affiliations:** Centro de Investigaciones en Geografía Ambiental, Universidad Nacional Autónoma de México (UNAM), Apartado Postal 27-3 (Santa María Guido), Morelia, Michoacán, 58190 Mexico; Escuela Nacional de Estudios Superiores Unidad Morelia, Universidad Nacional Autónoma de México (UNAM), Apartado Postal 27-3 (Santa María Guido), Morelia, Michoacán, 58190 Mexico; Instituto de Investigaciones en Ecosistemas y Sustentabilidad, UNAM, Universidad Nacional Autónoma de México (UNAM), Antigua Carretera a Pátzcuaro 8701, Morelia, Michoacán, 58190 Mexico

**Keywords:** Agroecology, Agroforestry systems, Sustainable management, TEK, Traditional management

## Abstract

Transformation of natural ecosystems into intensive agriculture is a main factor causing biodiversity loss worldwide. Agroforestry systems (AFS) may maintain biodiversity, ecosystem benefits and human wellbeing, they have therefore high potential for concealing production and conservation. However, promotion of intensive agriculture and disparagement of TEK endanger their permanence. A high diversity of AFS still exist in the world and their potentialities vary with the socio-ecological contexts. We analysed AFS in tropical, temperate, and arid environments, of the Tehuacan Valley, Mexico, to investigate how their capacity varies to conserve biodiversity and role of TEK influencing differences in those contexts. We hypothesized that biodiversity in AFS is related to that of forests types associated and the vigour of TEK and management. We conducted studies in a matrix of environments and human cultures in the Tehuacán Valley. In addition, we reviewed, systematized and compared information from other regions of Mexico and the world with comparable socio-ecological contexts in order to explore possible general patterns. Our study found from 26 % to nearly 90 % of wild plants species richness conserved in AFS, the decreasing proportion mainly associated to pressures for intensifying agricultural production and abandoning traditional techniques. Native species richness preserved in AFS is influenced by richness existing in the associated forests, but the main driver is how people preserve benefits of components and functions of ecosystems. Elements of modern agricultural production may coexist with traditional management patterns, but imposition of modern models may break possible balances. TEK influences decisions on what and how modern techniques may be advantageous for preserving biodiversity, ecosystem integrity in AFS and people’s wellbeing. TEK, agroecology and other sciences may interact for maintaining and improving traditional AFS to increase biodiversity and ecosystem integrity while improving quality of life of people managing the AFS.

## Background

Transformation of natural ecosystems into intensive agricultural systems is among the main factors determining the high biodiversity loss throughout the world [1–5]. The expansion of agriculture and grassland areas is progressively growing. For instance, from 1980 to 2000, more than 55 % of new agricultural fields were established in primary forests and nearly 28 % in secondary forests [6]. Main efforts for biodiversity conservation have been directed to decree natural reserves and protected areas, but these have only protected nearly 8 % of the planetary forests [7]. Most of the natural protected areas are surrounded by or have inside them agricultural landscapes, commonly with environmental problems such as fragmentation, contamination by agrochemicals, illegal hunting and taming, soil erosion, among others [8, 9]. Therefore, threatening of biodiversity within and outside protected areas are closely linked with socio-ecological processes occurring in landscapes at the surrounding area as well as inside them, including the matrix of agricultural and forest lands, and the management forms of agricultural systems [10–12].

In extent areas of the world, landscapes comprise agroforestry systems (AFS), which may play important roles for maintaining biodiversity and ecosystems integrity [2, 13]. These systems are strongly supported by TEK, ecology, and agroecology; therefore, cooperation of TEK and ecological sciences in understanding and acting may be crucial for optimizing such a role of AFS in conservation programs while ensuring the wellbeing of people managing the systems.

AFS are complex systems combining wild and domesticated plant, animal, fungal and microorganisms components interacting, determining processes and emergent properties with beneficial consequences for both ecosystems and societies. These systems are designed and managed based on millenarian experiences of peoples throughout the world and are expressions of TEK and biocultural heritage. Traditionally, management of AFS include among their components trees, shrubs and herbs that are part of the natural forests, which confer them good capacities for conserving native species. Native species may be let standing, both adult plants and their sprouts, but also people may plant their propagules, or provide them special care [14–16].

Components of AFS are managed in systemic ways of land use [17–19] and practices could be reminiscences of the earliest forms of agriculture [20]; actually, several authors have postulated that agriculture derives from experiences of managing forests [21]. Current AFS are apparently the result of TEK based on centuries and even millennia of interactions between humans and nature, as well as knowledge and techniques resulting from such interactions. AFS synthesize the expression of ancient and deep biocultural interactions and TEK [22–25].

AFS have been studied from ecological sciences [26, 27], economic [1, 28], cultural and ethnoecological approaches [22, 25, 29], landscape analysis [4], among other disciplines. It is generally recognized that AFS integrate multiple wild and domesticated plant and animal components [25] in a coherent totality [30]. Crops and forest components are disposed in dynamic patches determining a high diversity of biophysical and socio-ecological processes [23] that favour the conservation and resilience of both components and functions [11, 31–34].

From an ecological perspective, at landscape level AFS may conform corridors that favour processes of dispersion of native flora and fauna, as well as maintenance of ecosystem functions that provide valuable environmental services. Among them, carbon sequestration, refuge of pollinators and natural predators of crop pests, reservoirs of propagules of plants for vegetation regeneration, soil conservation, and regulating factors of water, nutrient flows, and microclimates [2, 5, 35–38].

It has been documented that soils of AFS may be productive enough to sustain long term agricultural production [13, 17, 33], and are highly beneficial to maintain and improve production in areas with soils of low fertility and high or low humidity [37]. Presence of a cover of wild and domesticated plants favours availability of nutrients like nitrogen and absorption of nutrients in deep layers of soil [5, 37, 38].

AFS may favour maintenance of local and regional biodiversity [2, 23]. At regional level, the mosaic of agricultural and forest patches allows maintaining habitats, connectivity and gene flow among populations of flora and fauna species of conserved and fragmented areas [9, 26, 27, 33, 39–43]. At local scale, AFS may increase the floristic composition of both useful and not useful plants species [44], wild, weedy and domesticated plants [14, 21, 45], species from primary and secondary forests, and even plant species from several forest types of a region [16, 45–49]. All these practices significantly contribute to increase the species richness and diversity in AFS and the surrounding landscapes. In addition, some components of AFS, particularly trees, provide habitats favourable to other plant (e.g. epiphytes and hemi-parasitic plants) and animal species [2, 13, 15]. These processes, in turn, favour the coexistence and interactions among species, the stability and resilience, as well as a more sustainable productivity of the system than monocultures [1, 26, 50, 51].

AFS favour the potential of regeneration of forests disturbed by establishing of agricultural plots, and ease its restoration and that of the neighbouring systems. While conserving soil, water, animals dispersers of seeds, pollinators, and propagules of native plant species, AFS allow that fallow and abandoned agricultural plots are in good conditions for a faster succession and regeneration of natural ecosystems than those systems drastically transformed [9, 52, 53].

AFS have been used based on the principle of multiple use of natural resources and functions of the systems, which has favoured the diversification of production systems for rural people subsistence [22, 28, 54]. The strategy of multiple use of resources prevail in rural peasant contexts, mainly where traditional agriculture is predominant, and where people have a close relationship with their land and perceive and know its multiple constituents, functions and interactions [9, 22]. Such management strategy allows that AFS provide agricultural products along with forest resources used as construction, fuel, medicines, food, and other benefits [1, 26, 47, 49, 55, 56], which in turn may contribute to decrease pressure on resources extraction and clearing of forest areas. In addition, these have allowed generating monetary incomes to households in different regions [47, 48].

However their agroecological and biocultural importance, AFS are in high risk of disappearing. Among the factors that mainly endanger their existence, are the increasing social and economic pressures to intensify the production systems [25, 57], as well as loss of TEK associated to loss of traditional cultures, knowledge and techniques [22]. In addition, in Mexico, some governmental programs enhance removing vegetation cover in crop fields arguing that the remaining vegetation decreases agricultural production [15, 25]. Some programs of governmental agencies and NGOs are careless, enhancing reforestation or AFS without considering local knowledge and opinions. These programs are generally unsuccessful [28, 54, 58]. Documenting TEK and management experience associated to AFS throughout the World is therefore a high priority of ethnobiological and agroecological research.

AFS have persisted throughout the time from the origins of agriculture. In the Tehuacán Valley, one of the earliest areas of agriculture of the New World [59], AFS predominate in the regional agricultural landscape [15, 47] and are most probably the earliest agricultural systems in that region, and probably in other areas of Mesoamerica. The hypotheses include the “hydro-horticulture” and the “streams horticulture” proposed by MacNeish [59], the forest disturbance model proposed by Smith [60], and the silvicultural origin model proposed by Casas et al. [14, 21]. A high diversity of AFS has been reported in the Tehuacán Valley, Mexico and other areas of the world [2, 5, 25, 27, 38, 41]. Their potentialities and limitations varying according to the ecological, cultural, social and economic contexts where they are practiced. Our study aimed to examine how social and ecological factors influence the management and structure of AFS systems. In particular, we directed our review to analyse how variable are AFS systems in different environmental contexts in Tehuacán Valley, how varies their capacity to conserve biodiversity, and which factors mainly influence the variation. We hypothesized that biodiversity harboured in AFS should be proportional to amount of diversity existing in the associated forests, as well as to the prevalence of TEK and management over intensive agricultural systems.

## Methods

**Fig. 1:**
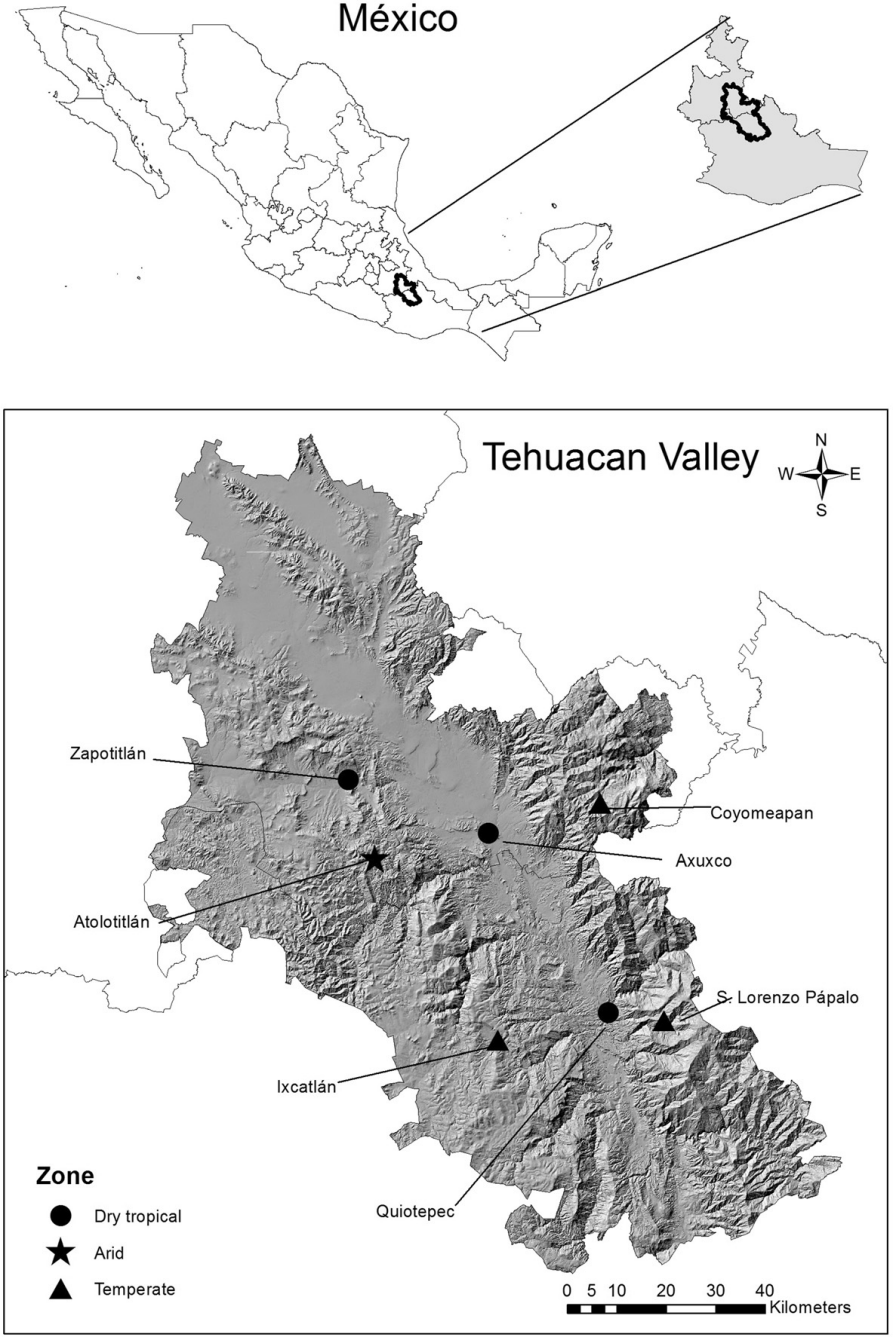
Location of the Tehuacan Valley in Central Mexico. We indicate the names of the localities where the study was conducted and the type of environment studied

We conducted studies in a matrix of environments and human cultures in the Tehuacán Valley, central Mexico (Fig. 1) during nearly 10 years of studies comparing the vegetation composition of AFS and forests. Such studies have included AFS and different forest types in (1) dry areas (columnar cacti forests, thorn-scrub, and rosetophyllous forests), (2) tropical sub-humid and dry forests, which are associated to the rivers conforming a gradient from tropical dry forests to real tropical wet forests, and (3) temperate zones (oak-pine forests, sclerophyllous forests or mexical a type of Mediterranean-like vegetation). AFS systems may include two great groups of systems, those attached or close to peoples’ houses (homegardens), and a great variety of systems apart from houses we call them in this study “field AFS”. “Field AFS are not only different to homegardens in relation to the distance from houses but in relation to their function in production, extent, and capacity for maintaining natural vegetation. Our studies are now centred in “field AFS”, and a comparison with homegardens will be discussed elsewhere. The analyses based on vegetation sampling, which allowed calculating the species richness, diversity and composition, relating density, frequency and biomass of each species in the sampling areas in both, forests and AFS. Plant composition was evaluated through the number of plant families, genera and species, considering all species and only native plant species. In forests we sampled square units of 500 m^2^ subdivided in five squares of 10X10 m^2^. For sampling AFS we recorded individual plants of each species in areas of vegetation cover associated to agroforestry practices (remaining patches of forests inside agricultural plots, vegetation islands, vegetation fringes, isolated trees and living fences surrounding agricultural plots). In addition, we have documented general features of local human cultures, their agricultural knowledge and practices, emphasizing about the reasons why people maintain vegetation cover in their agricultural plots, the economic, ecological and agricultural benefits perceived by practicing AFS. We compared plant species richness and diversity among forests and AFS, as well as among AFS managed at different levels of intensity.

In addition, the studies included interviews to the owners of each agricultural plot that allowed documenting the type of management, land tenure, managed crops, the destiny of production (direct consumption, commercialization or interchange), total amount of production, use or not of machines, agro-chemicals, as well as strategies and techniques of management of natural vegetation maintained inside and around the plot, the purpose and reasons for maintaining the particular components.

Our study includes also information systematized from different regions of Mexico. During the last five years, we have compiled and systematized ecological, agronomic, human cultural and economic information on AFS of Mexico. Consequently, we have constructed the database “Biblioteca de Sistemas Agroforestales Tradicionales de México” (Library of Traditional Agroforestry Systems of Mexico), leaded by Ana Isabel Moreno-Calles, which can be consulted at https://www.zotero.org/groups/sistemas_agrofo-restales_tradicionales_de_méxico/.

We finally conducted an exhaustive review of literature on agroforestry systems from tropical, temperate and dry areas of the world, centring our attention on information comparing biodiversity conservation and ecosystem services in the context of traditional and intensive agricultural management in order to compare and analyse the context of our own information.

## Results

### Agroforestry systems in tropical areas

The most studied AFS are those located in tropical regions [2, 53, 61–64]. In the Tehuacán Valley, México, the tropical areas are located throughout the main permanent rivers and numerous streams of the region. There is a gradient of tropical vegetation, from tropical dry forests in the northern zone of the region to sub-humid vegetation represented by mezquitales in the central portion of the alluvial valleys to tropical wet forests in the lowest southernmost areas, where forests are really tropical rain forests surrounded by dry areas. Our studies documented mainly those sub-humid areas [49] that in lowland alluvial valleys. In these tropical forests zones, AFS associated to the multi-crop traditional system called milpa (commonly cultivating maize, beans and squashes) maintain 66 species of trees and shrubs, 81 % of them being native species natives and representing nearly 38 % of the woody flora of the forests surrounding the AFS (Tables 2 and 5). We found that even some endemic species may increase their abundance with agroforestry practices.

#### Agroforestry practices

In the tropical area, the most important agroforestry practices are semi-terraces, with fringes containing living fences and shrubby and herbaceous vegetation, where people maintain wild species. In some cases people move individual plants that are inside the plots to these fringes, which contribute to maintain soil and humidity and prevent erosion. The reasons for maintaining natural vegetation inside agricultural plots are mainly utilitarian, since most of them are used as food (fruits, the greens called quelites), medicinal, fodder, fuelwood and wood. However, it is also explicit the purpose of preventing soil erosion, maintaining soil fertility and having shade for resting during the agricultural work. In addition, people commonly say that they maintain trees and shrubs to ensure the specific hábitat of some other useful species. For instance, they maintain *Parkinsonia praecox* since the edible larvae *cuchamá* live on their stems and branches. Another example is the mezquite (*Prosopis laevigata*), which is maintained as support for pitahaya (*Hylocereus undatus*). Other non-utilitarian reasons are related with the beauty that plants provide to the plot, rituals, respect to use regulations, prestige or simply the recognition of the right of plants to live. We recorded 15 different reasons (Table 1).

**Table 1:** Main reasons why people from different socio-ecological zones of the Tehuacán-Cuicatlán Valley, central México, let standing trees and shrubs in their agroforestry systems (AFS)

	Reasons	Temperate zones	Arid zones	Tropical dry and wet zones
Utilitarian	Edible product	X	X	X
	Firewood	X	X	X
	Fodder	X	X	X
	Edible fruit	X		X
	Tools	X	X	X
	Medicine	X	X	X
	Timber	X		
	Construction		X	
	Beverages	X	X	
Ecosystem benefits	Shade	X	X	X
	Maintenance of fertility	X	X	
	Erosion control		X	X
	Water control			X
	Windbreaks	X		X
	Attractor of rain	X		
	Boundary	X	X	
Crop management	Support climbing crops	X		
	Habitat of useful species		X	X
	Storing straw		X	
	Conforming the terrain			
Ethics	Part of nature	X	X	
	Ornamental			X
	Ceremonial		X	X
	Does not affect	X		X
	Use regulations			X

In the humid tropical areas of Mexico, some outstanding AFS are found in south-eastern Mexico, where Ford and Nigh [65] and other authors identified that the shifting agriculture practiced by the Maya peoples involve the management of nearly 70 domesticated plants species that are cultivated in the fallow areas, together with useful wild plant species. These systems are associated to wetter tropical forests than those found in the Tehuacan Valley. Management techniques involve cultivation, transplantation and which increase their abundance artificially in the recovered forests. This practice appears to be ancient not only in the Maya region, and not only in tropical forests but in other Mesoamerican areas and vegetation types [14, 21]. Another group of tropical AFS is represented by the *Kuojtakiloyan*, a system studied in the Sierra Norte of the state of Puebla by Toledo and Moguel [66]. This system is also wetter than the tropical sub-humid forests that we documented in the Tehuacan Valley. *Kuojtakiloyan* are managed by the Náhuatl people, and it is mainly directed to produce coffee under shaded conditions, but people maintain in the systems up to 140 plant species, nearly 96 % of them deliberately maintained because of their use. Together with such a high plant diversity, other associated biodiversity has been reported, mainly birds, amphibians and insects. The *Kuojtakiloyan* is representative of several similar systems practiced in tropical areas of Mexico by traditional peoples. It may be associated to cocoa, pineapple and other fruit production trees plantations, or other main crops, determining monetary benefits. However, these crops are commonly in association with multiple resources, retaining the capacity of maintaining high biodiversity and ecosystem services.

Several studies have reported that these systems may maintain on average nearly 60 % of species of plants, birds, insects and mammals recorded in the neighbouring forest zones [27]. Consequently, AFS are particularly important in the conservation policies of these areas, which are the reservoirs of the highest biodiversity and indigenous cultures of the whole planet [41]. Paradoxically, the tropical zones are also considered the most strongly disturbed areas on Earth [67, 68]. Approximately 70 % of their plant cover has been converted into agriculture or grassland areas [26], which has determined local extinction of numerous species and threatening tropical biodiversity [69]. The natural habitats are progressively decreasing; for instance, according to FAO [70] in Mexico and Central America the deforestation rate is nearly 1.2 % of the total cover per year, which proportionally increases fragmentation, severe degradation and pressure on the remaining forest [33].

In numerous tropical landscapes, AFS are the managed ecosystems more similar to natural forests [5]. During the last decades, agroforestry has promoted in tropical areas strategies to manage in balanced ways natural resources of forests in association with agriculture, water and soil management, and biodiversity conservation [9, 71] together with efforts for the maintenance of ecosystem services [33, 37]. Such holistic approach of ecosystem management has resulted in successful practices for conserving biodiversity, ecosystems and their capacity for recovering after disturbance. Removing trees from AFS may determine the reduction of resistance and resilience of the agricultural system and the household units that manage them, increasing the incidence and vulnerability to pests and climate change [4, 5]. Therefore, the maintenance of these elements in the systems becomes critical for sustainability of the natural and artificial systems, as well as the households’ life.

In the tropics of the world, there are numerous examples of AFS of coffee and cocoa plantations favouring conservation (Table 2), but AFS combining production of staple grain crops combined with fruit trees are also common. These systems have also demonstrated to be relevant in biodiversity conservation, maintaining high levels of species richness, diversity and structure similar to those of cocoa AFS referred to above [63, 72, 73]. Studies by Asase and Tetteh [74] confirm that trees maintained in these systems provide shade, reduce evapotranspiration, erosion and destructive effects of strong wind (Table 2).

**Table 2:** Examples of species richness and diversity maintained in agroforestry systems (AFS) in different tropical, temperate and arid zones of the World

Reference	Environment	Region	AFS	Species richness	Diversity	% of species from forest	Taxa evaluated
Bhagwat et al. (2008) [27]	Tropical	General review	General review	N/A	N/A	60 %	General
Toledo and Moguel (2012) [66]	Tropical	Puebla, México	Coffee plantations	140	N/A		Plants
Steffan-Dewenter et al. (2007) [68]	Tropical	Central Sulawesi, Indonesia.	Cocoa plantations	189 trees		40 %	Trees
				166 herbs	N/A		Herbs
				208 insects		40 %	Insects
Sonwa et al. (2007) [91]	Tropical	Central and southern Cameroon,	Cocoa plantations	203	Sørensen 0.44		Trees
					Shannon 3.7		
					Simpson 0.12		
						70 %	Ants
				30	N/A	N/A	Ants
Asase and Tetteh (2010) [74]	Tropical	Adjeikrom, Ghana	Cocoa plantations	27	Shannon 2.46	N/A	Trees
			Other crops	31	AFS 2.6 Forest 4.94	N/A	
Okubo et al. (2010) [63]	Tropical	Java, Indonesia	Bamboo gardens	76	Shannon: 1.66	N/A	Trees
Vallejo et al. (2014) [49]	Temperate	Tehuacán Valley, Mexico	Maize fields	79	N/A	43 %	Trees and shrubs
Moreno-Calles et al. (2010) [15]	Arid	Tehuacán Valley, Mexico	Maize fields	73	N/A	59 %	Plants
Nabhan (1987, 2007) [24, 54]	Arid	Northern Mexico	Cultivated oasis	139	N/A	N/A	Plants
				103	N/A	N/A	Birds
				14	N/A	N/A	Mammals
Blanckaert et al. (2007) [45]	Arid	Tehuacán Valley, México	Maize fields	161	N/A	49 %	Herbs

N/A The information was not available in the revised article

A common problem in AFS of the tropical regions is the increasing of mechanization and use of agrochemicals, which has in turn favored increasing of pests problems [1, 67, 68] (Table 4). The industrialization of agriculture and public policies favoring intensive agricultural systems have been a main cause of transformation from diversified traditional agriculture to agro-industrial systems highly dependent from agrochemical inputs [9, 72, 75].

### AFS in temperate zones

**Fig. 2:**
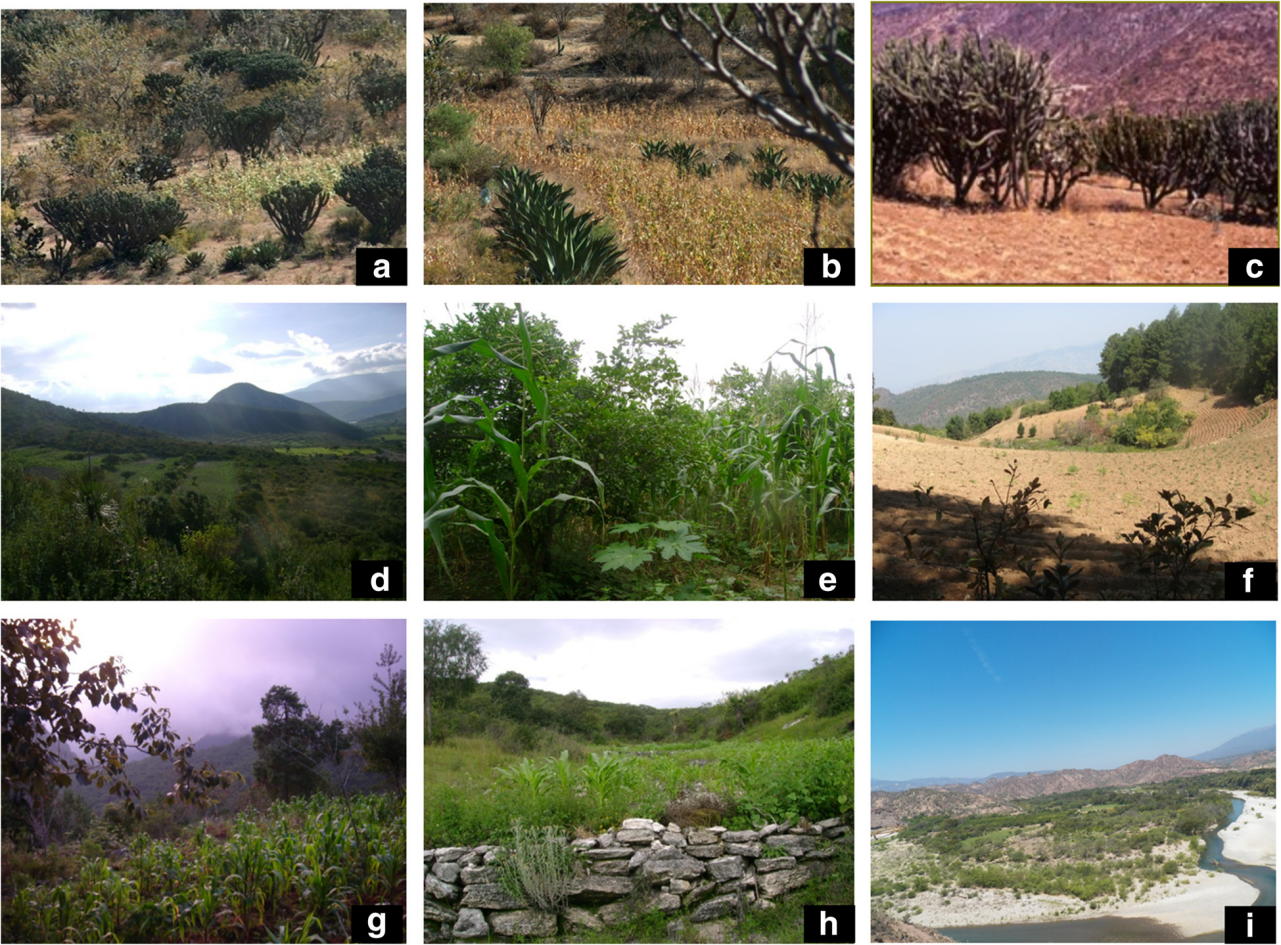
Mexican ethnoagroforestry systems, integrating elements from forests (wild) and agricultural or domesticated components of biodiversity in the Tehuacan Valley. The image illustrate several agroforestry practices such as friges of vegetation, isolated trees, terraces, and barriers against strong wind. AFS of the Tehuacán Valley showed in this image represent different environments discussed in the text but cultivation of maize and beans are the main annual crops in all cases, changing the composition of the wild vegetation composing the system. **a** AFS with *Polakia chichipe* and *Myrtllocactus schenckii* in the arid zone studied, **b** Fringes of vegetation mainly composed with *Agave salmiana* in the arid zone studied, **c** AFS with patches of vegetation composed by *Escontria chitilla*, *Myrtillocactus schenckii*, *Polaskia chchipe* and *P. chende* in the arid zone studied; **d** Fringes of terraces in the sub-humid region studied; **e** combination of native and domesticated trees, crops and herbaceous species; **f** Example of the aspect of an Island of vegetation in a cleared cropland; **g** Terraces constructed with ground and plants, **h** Terraces with stones, **i** General aspect of the landscape of AFS associated to the river

Temperate zones have demonstrated high potential for favoring long-term sustainable management practices of AFS and natural resources use [20, 76, 77]. In the Tehuacán Valley, we recently documented [49] that AFS associated to temperate forests are mainly managed with the multi-crop traditional system called milpa (commonly cultivating maize, beans and squashes) maintaining 79 species of trees and shrubs, 86 % of them being native species native to the region, representing nearly 43 % of woody flora of the forests surrounding the AFS (Tables 2 and 5). Local people said that the main reasons for maintaining trees and shrubs in their agricultural plots are their use as fruit trees, fuelwood, shade, protection of annual crops against wind, beauty, and respect to nature, among other purposes (Table 1, Fig. 2).

AFS in these areas may be as ancient as in the tropical zones not only in the Tehuacán Valley but also in Mexico. In the temperate zones of the Americas, there are abundant records of old and recent annual crop systems of maize cultivated in combination with native trees including pines, oaks and other wild species, as well as fruit trees such as apples, pears, plums, among others [78]. In these areas there are specific problems such as climatic and microclimatic conditions such as snow, hailstorms, and frosts, maintaining of soil nutrients, control of underground water level, maintenance of favorable habitats for plants, insects and other animal species, fungi and microorganisms, all of them representing challenges for agricultural practices; stabilization of soils protection against wind are among the most common factors motivating maintenance of natural vegetation in AFS of this zones [79] (Table 3). The most common agroforestry practices in temperate zones are the combination of annual crops with fruit and timber producing trees, frequently forming terraces, as well as a great variety of practices for establishing fences for protecting plots against strong winds, soil erosion, and other multiple purposes (Table 3).

**Table 3:** Ecosystem benefits reported for agroforestry systems from tropical, temperate and arid zones

Reference	Environment	Region	SAF	Ecosystem benefits
DeClerck et al. (2010) [33]	Tropical	Mesoamerica	General review	Biological corridors enhance secondary succession, favour pollination, biological control of pests, maintenance of microclimates, favour restoration.
Tscharntke et al. (2011) [5]	Tropical	Tropical areas of the World	Cocoa and coffee plantations	Provide biomass for construction, fuel and food (fruit). Favour soil fertility stimulating decomposition of leaf litter, the nutrient cycles and erosion control. Increasing carbon sequestration and reduction of GEG emitions, contributing to mitigation of effects of climate change. Improve functional biodiversity.
Bhagwat et al. (2008) [27]	Tropical	Tropical areas of the World	General review	Refuge of species out of protected areas, maintain heterogeneity of habitat and landscape, reduce anthropogenic pressure on wild areas, may be buffer or corridor areas among conserved zones.
Steffan-Dewenter et al. (2007) [68]	Tropical	Central Sulawesi, Indonesia.	Cocoa plantations	Reduce soil temperature, increase humidity and decomposition rate and maintain soil fertility.
Jose et al. (2004) [79]	Temperate	General review	General review	Biological control through ecological interactions among species, nitrogen fixation due to presence of legume species.
Quinkenstein et al. (2009) [76]	Temperate	Europa	General review	Improve microclimates and thus the stability of productivity of crops. Efficient use of hydric resources and nutrients, sustainable use with low input of fertilizers, pesticides and hand labour. Increase structural heterogeneity of landscape. Promotion of biodiversity, favoring landscape connection. Carbon capture in soil.
Vallejo et al. (2014) [49]	Temperate	Tehuacán Valle, México	Maize fields	Provide shade, protect against strong winds, and provide fruit. Fuel wood and fodder.
Shankarnarayan, K. A., Harsh, L. N., & Kathju	Arid	India	Prosopis AFS	Stabilize dunes, maintain trees adapted to thin soils, reduce effects of strong wind, reduce evapotranspiration, multipurpose trees (fruit, fuel, fodder, etc.).
Moreno-Calles et al. (2010) [15]	Arid	Tehuacán Valley, México	Maize fields	Provide shade, fodder and fruit
Altieri and Toledo (2005) [22]	Arid	México	General review	Conservation of soil and water, reduce evapotranspiration, maintain soil fertility and useful species.

The diversity and structure of plant components of AFS in these zones allow to regulate microclimatic conditions, to favor moderate temperatures, reducing intensity of solar radiation and improving the water (decreasing effects of frosts, snow and storms, increasing the humidity and buffering erosion effects of rain and wind, and reducing the loss of evaporation of superficial water). Although trees may compete for water with crops, benefits are higher [76].

#### Agroforestry practices

Living fences were identified as the main and most extent agroforestry practice. In these fences people maintain the greater number of individual plants of wild vegetation, and are mainly directed to protect plots against strong wind. Another important practices are vegetation islands, which are arranged in small vegetation patches that allow maintaining natural vegetation which is let standing because of their use and ecosystem services without hindering agricultural labors such as passing through of ploughs or even tractors. In these patches it is common to find domesticated fruit producing trees together with several native species let standing with several purposes. Vegetation patches are mainly left growing in areas of the plots where it is difficult to practice agricultural activities; for instance where stones are abundant, the topography particularly pronounced flooded areas or inappropriate soils. In these areas people commonly use to let standing growth and abundance of a great variety of species of natural vegetation, particularly in areas that do not affect agricultural practices.

Temperate zones generally have high availability of water, enough for allowing production of domesticated trees, and sometimes more than one season of production of annual crops. The main reasons for maintaining vegetation in AFSs of this region are food benefits, but we identified a total of 16 reasons mentioned by people (Table 1).

AFS of temperate zones have been documented as important reservoirs of native biodiversity; on average, agroforestry systems harbor two to three times more species than intensive systems [80]. It may also include a high number of species and crop varieties specifically adapted. For instance, nearly 10,000 varieties of apples and 1000 to 2000 varieties of plums are maintained in situ in these systems [81].

In areas of AFS of temperate zones the heterogeneity of landscapes has being decreasing with agro-industrialization, a phenomenon that is more pronounced in developed countries, but even in those zones AFS have adapted to changes, maintaining and providing ecological structure for species inhabiting the agricultural landscapes [82] (Table 4). These landscapes provide essential services for human wellbeing [76, 81].

**Table 4:** General advantages and problems of agroforestry systems of tropical, temperate and arid zones

Ecological zone	Uses	Main agroforestry practices	Ecosystem benefits	Problems
Tropical	• Shade crops (coffee. cocoa)	Fringes	Soil protection	Intensification
	• Fruit production	Living fences	Shade	
	• Timber products	Isolated trees	Soil fertility	Loss of TEK
	• Pest control		High biodiversity	Pesticides, deforestation
Temperate	• Windbreaker	Windbreaker barriers	Microclimate conditions	Intensification
	• Snowing damage	Living fences	Buffering winds	No replacement of trees
	• Fruit production	Fringes, isolated trees	Soil protection	Fruit commercialization
	• Timber products	Vegetation patches	Reduction of damages by frosts	Loss of TEK
	• Control of pests	Vegetation patches	Biodiversity	Industrialization
Arid	• Resistance to dryness	Windbreaker barriers	Water management	Specificity of native species
	• Retention of humidity and soil	Isolated trees	Soil protection	Abundance of rare species
	• Timber and non-timber	Vegetation patches	Shade	Intensification
	• Medicinal	Living fences, fringes	Biodiversity	I

### AFS of arid zones

Arid zones are characteristic for low rainfall, high radiation and extreme temperatures. These conditions determine high hydric stress and biotic communities of these areas have particular adaptations, highly specialized to survive in such extreme conditions [83]. Ecosystems of these zones are particularly fragile, and relatively small disturbances may determine great consequences including the irreversible loss of components [69], such loss may be more drastic than in other ecosystems [83–86]. Production activities have required fine and deep ecological knowledge and management practices according to particular ecological, cultural, and social components [86]. In order to minimize the negative effects associated to environmental conditions, local peoples of the arid lands have developed production systems in which the woody perennial species have an important role in terms of production and conservation [87].

Several studies illustrate the capacity of AFS of arid zones for conserving biological diversity. In the Tehuacán Valley, AFS are managed at small scale, mainly associated to the multi-crop called milpa, with low use of chemical inputs and mechanization, and most commonly their products destined to direct consumption by households [47]. Several studies conducted in the area (Table 4), reveal that AFS play an important role to satisfy human needs and conserving biodiversity in terms of species richness and genetic diversity of particular species [88–90]. Moreno-Calles et al. [15, 47] reported that on average 59 % of plant species of natural forests are maintained in AFS, and that some endemic species such as *Escontria chiotilla* and *Lemaireocereus hollianus* may increase their abundance in these systems. Blanckaert et al. [45] found nearly 161 of herbaceous plant species in agricultural systems of the area, nearly 49 % of them being present in the local forests (Tables 1, 2 and 5). Dominant arborescent cacti species of the region such as *Stenocereus stellatus*, *S. pruinosus*, *Escontria chiotilla*, *Polaskia chende* and *P. chichipe* have been reported in AFS to have on average 93.8 % of genetic variation occurring in wild populations. But in some plots were recorded with even higher levels of genetic diversity than in wild populations [12, 15, 88]. It is relevant in addition to mention that populations of the species mentioned in AFS maintain a dynamic gene flow with wild populations, which also indicate that AFS are key areas for implementing policies of regional biodiversity conservation.

**Table 5:** General socio-ecological and technological aspects recorded in AFS in different indigenous and Mestizo communities of the Thuacán Valley, México

		Temperate			Arid	Arid-alluvial valleys		
Aspect	Variable	Santa María Coyomeapan	San Lorenzo Pápalo	Santa María Ixcatlán	San Luis Atolotitlán	Santiago Quiotepec	Zapotitlán Salinas	San Losé Axusco
Sociocultural	Ethnicity	Náhuatl	Cuicateco	Ixcateco	Mestizo and Náhuatl	Mestizo and Cuicateco	Meztizo and Mixteco	Náhuatl
	Land tenure	Private, ejidal and communal	Communal and private	Communal	Ejidal	Communal and private	Communal	Communal and ejidal
Farming practices	Crops	Corn, beans, squashes, pumpkins, peas	Corn, bean, gourd, fava beans, peas	Corn, beans and squashes	Corn, beans and squashes	Corn, beans and squashes	Corn, beans and squashes	Corn, beans and squashes
	Fallow	1 year	1–3 years	6 months	1–5 years	6 months	6 months	6 months
	Irrigation	No	No	No	No	Yes	Yes	Yes
	Machinery	Mattock	Plough and mattock	Tractor and mattock	Plough	Plough	Tractor and mattock	Tractor and Plough
	Agrochemicals	No	Yes	No	No	Yes	Yes	No
	Livestock	Yes	Yes	Yes	Yes	Yes	No	Yes
Agroforestry systems	Agroforestry practice	Boundaries, windbreaks and isolated trees	Boundaries and vegetation islands	Boundaries and patches of vegetation	Isolated trees, vegetation islands and fringes against soil erosion, vegetation surrounding the fields.	Fringes for water managenment and against soil erosion	Fringes for water managenment and against soil erosion	Fringes for water managenment and against soil erosion
	Principal uses	Edible fruit, Other edible product and Firewood	Shade, firewood and boundary	Shade, firewood and boundary	Edible fruit, firewood and shade	Edible fruit, shade and fodder	Asthetic (beauty), shade and Maintaining water	Other edible product, shade and fodder
Ecological	Species richness	39	18	29	71	32	58	10
	Diversity (Shannon Índex)	3	2.5	2	3.2	N/A	N/A	N/A

#### Agroforestry practices

In the AFS of the arid zones of the TCV, we documented several managemet practices, such as vegetation islands and patches, living fences and isolated trees. The latter agroforestry practice is represenred bt arboreaus species of great economic and cultural value for local people. These include big trees providing shade, fruits, fuelwood and fodder. In arid zones of the TCV the main reason to maintain native vegetation in agricultural plots are shade, fodder, and other 13 reasons (Table 1).

In other regions of Mexico, in the Sonoran Desert for instance, Nabhan [54] documented the traditional agricultural techniques practiced by the Papago people, who have conserved the oases of their territories and have developed a complex system of biotic interactions. This author identified eight plant associations and various agroforestry practices including living fences and windbreaker barriers, as well as high levels of diversity of trees, birds and mammals. The Papago have modified the landscape geomorphology through terraces, channels and flood zones [22, 91]. In the Mezquital Valley in central Mexico, the Ñañhú or Otomí people have constructed terraces and borders to manage water and sediments to improve soil and humidity for crops. Particularly important for these purposes are agave, which in addition provide multiple products used as food, beverages, and fibers, and others [22] (Table 3).

In the arid zones of the numerous human cultures have interacted with the difficult conditions of these zones for thousands of years, and a significant amount of knowledge and techniques have been developed [22, 24], which are all crucial at present for designing the future. Investigating trees and shrubs associated to crops may provide valuable information for improving the AFS, conserving biodiversity and supporting techniques for restoring disturbed areas of arid zones [24, 47] (Tables 3 and 4, Fig. 2).

## Discussion

### TEK and biodiversity and ecosystem management

Our studies in the Tehuacán Valley reveal that the bases of variation of practices and management techniques at both particular elements of biodiversity and ecosystem levels is the presence, explicit expression and depth of traditional ecological knowledge. In that region, we have documented more than nearly 2000 species of plants that are used with a high variety of purposes. Local people know their useful properties but also details about their distribution, abundance, interactions with other plants and animals and they have an extraordinary information about germination, growth and phenology. All these elements are crucial expressions of TEK that significantly influence their decisions about what and how managing in AFS. Blancas et al. [46] documented with an extraordinary detail how people make decisions about which edible plants may or may not be managed, should or should not be managed, and how and in which systems would be managed. These authors show that TEK is an on-going construction, continually adapting to the changing conditions. The new conditions, as the authors discuss, may be ecologically, economically, culturally and technically influenced. And all these elements may be highly dynamic.

Our studies in the Tehuacán Valley (Table 5) allow seeing that indigenous peoples may adopt technologies of intensive agriculture, and they decide how necessary and viable their adoption is. Ethnicity may or may not be representative of the maintenance of traditional agricultural practices or the adoption of modern technologies. For instance, Table 5 shows that in Axuxco, an eminently Náhuatl village, people decided the adoption of modern elements of agriculture (tractor and agrochemicals), whereas in Santa María Ixcatlán, where the Ixcatec people are markedly few and Mestizo people is the great majority, the traditional agricultural practices in AFS are clearer. Another important aspect to mention is that adoption of modern agricultural techniques not necessarily is the cause of decreasing vegetation cover and biological richness and diversity maintained in AFS. For instance, in Table 5 it can be seen that some communities using tractor and agrochemicals may also maintain a high vegetation cover and plant diversity inside their plots. Therefore, causes and effects of traditional management and intensive agriculture are not linear in relation to ethnicity and vegetation cover, respectively. These are aspects that deserve a deeper analysis, which is of great importance for constructing policies in relation to agricultural patterns and biodiversity conservation.

Literature available on this and other issues reviewed in this analysis has been produced from different approaches and an appropriate comparison and establishing of general conclusions is for the moment difficult. However, further research on AFS including explicitly the questions of how traditional societies adopt and adapt modern agricultural elements and how much these elements influence decreasing biodiversity conservation capacity of AFS are of high importance for constructing alternatives and policies at different scales.

### Particularities of AFS in specific environmental contexts

Local peoples manage AFS according to environmental particularities in order to make them functional, productive and viable. In tropical zones, AFS multi-crops are commonly associated to let standing or planted components of the exceptionally diverse natural forests. Coffee and cocoa plantations are out-standing AFS in terms of biodiversity conservation. Soil degradation due to rain and deforestation are main problems faced by people through conserving high species richness, mainly of fruit and fine timber trees but also numerous herbaceous plants. In temperate zones, the main challenges are erosion caused by rain and wind, as well as loss of production caused by frosts, snow, storms and wind. In these areas, the windbreaker barriers, terraces and borders, as well as isolated trees contribute to create propitious microenvironments in AFS, particularly through trees providing fruit, fuel and wood. In the arid zones, the environmental pressures are mainly associated to drought and erosion caused by wind and the infrequent but heavy rains. These zones are particularly fragile since removal of particular species may determine drastic alterations in biotic interactions (pollination, seed dispersal, and facilitation of establishment of numerous plant species by nurse plant species), which may be particularly sensible because native species of perennial plants generally have slow growth rates. AFS in these areas are designed and managed in order to attend these problems and to optimize and ensure availability of valuable natural resources, as well as procuring ecosystem benefits associated to humidity, shade, propitious microenvironments and soil conservation (Tables 3 and 4).

### How does capacity of biodiversity conservation changes with environmental contexts?

The highest species richness of AFS have been reported in tropical areas, which is consequence of the also high natural species richness recorded of these ecosystems; however, when considering the proportion of species maintained in AFS, the ciphers are similar in the three environmental zones analyzed (Table 2). This result suggests that biodiversity conservation capacity is not an exclusive function of the nature of ecosystems.

The diversity of strategies for practicing AFS is high, and these patterns are associated to the diversity of agricultural techniques developed among ecological zones as well as regions of the World. It is difficult with the information available for the moment establish general conclusions. Every region has environmental socio-ecological and biocultural particularities, and all of them influence the responses and strategies practiced by human groups. AFS are ancient systems of agricultural production and most probably the earliest forms of agriculture. Therefore, the experiences accumulated during thousands of years have influenced the diversity of technical responses.

Today, inventorying the diversity of strategies is particularly important to enhance the option of AFS as alternative of raw matter production for food and other industries based on more environmental friendly principles. Intensive agriculture has been highly criticized since it has required high amounts of water, machines, oil-based energy and toxic agrochemicals. Agricultural production based on organisms genetically modified has demonstrated not to be a viable solution to the environmental problems initiated during the green revolution but, contrarily, the possible cause of even higher socio-ecological problems. Therefore, the construction of alternatives should be based on the valuable millenary experiences of humans throughout the world that have been able to conceal conservation of biodiversity and ecosystems with the satisfaction of raw matter required for diverse industries. Some few companies producing seeds and agrochemicals are the main beneficiaries of the intensive technological alterative production models, but not most of the agricultural producers of the World, who, for the contrary are in risk of make stronger their dependent relations with such companies and making even deeper de poverty and inequity prevailing at planetary scale. This fact should motivate reflexions of humans to other models of future perspectives, based more strongly on the traditional ecological knowledge and management techniques, which have sustained the humanity for nearly 99 % of the history of humans as agriculturalists. Consequently, intensification of studies of strategies and techniques of AFS throughout the World should be a priority of research for several scientific disciplines, outstandingly ecology, agroecology and ethnobiology.

## Conclusions

AFS have been used since the origins of agriculture, and have maintained a millenary richness and ecosystem functions that are agroecological lessons for rescuing and constructing technological innovation for sustainable agricultural management and landscape maintenance and recovering. AFS summarize crucial biocultural heritage. AFS have important advantages for conserving biodiversity and ecosystem functions while satisfying human needs, at different spatial scales.

Technical experiences varied extraordinarily in part associated to the characteristics of the ecosystems where agriculture is conducted, but also according to human culture and history in the regions of the world. A global research strategy directed to analyse agricultural techniques, soils, productivity, and resilience capacity of the systems, biodiversity conservation challenges, among other main topics in different ecological and cultural contexts is a priority. The construction of alternatives technically and culturally viable would be possible based on such research strategy.

Contribution of such a research strategy would be a valuable source of alternatives more environmentally friendly than intensive agriculture. Each region of the world has a high variety of ecological and cultural conditions and human experience has also been highly variable. Therefore, efforts of such a global strategy require the participation of different disciplines. However, the role of ecology, agroecology and ethnobiology are crucial. Traditional ecological knowledge associated to agroforestry systems synthesize thousands of years of experience of managing biodiversity and agroecosystems, and this knowledge is a key stone for shortening times of building viable alternatives.

The results systematized in this study may not be representative of the situations throughout the world. Such analysis requires systematizing the broad experiences and knowledge documented, which is undoubtedly a priority task for ethnobiological and agroecological sciences. Documenting the techniques and purposes for maintaining wild plants together with crops, the richness and diversity biological components of the systems resulting of such intentions, the economic advantage of those actions and their ecological benefits are crucial aspects for designing sustainable strategies of management of these systems.

## Abbreviations

AFS, Agroforestry systems; FAO, Food and Agriculture Organization; TCV, Tehuacán-Cuicatlán Valley; TEK, Traditional ecological knowledge
